# Supplementary material for: Chromothripsis during telomere crisis is independent of NHEJ, and consistent with a replicative origin
Source: Genome Res. 2019 May;29(5):737–49. doi: 10.1101/gr.240705.118 (PMC6499312; doi:10.1101/gr.240705.118)
Supplement: Supplemental Material [file supp_gr.240705.118_Supplemental_file_1.zip › contigs/annotated_contigs/DB96/contig.2.DB96_length_568_mean_cov_18.3521126761.docx]

**DB96_length_568_mean_cov_18.3521126761**

```
TTCCTGGGATTCCCCATTGGAATATGCTGTATTAAAGCCCACTTAGTGCTTACCTAGAACAGTGCCTGGCACAGGTAAGCACAAATACA
 >chr1:28836926-28837225 - E=3e-168
ATTGCTTTAGGAAGATGCCTTTTTTTTGAGACTTAACTCTGTCACCAGGCTGGAGCGCAGTGGCAAGGTCTCAGCTCAGTACAACCTCC

ATCTCCCGGGTTCCAGCAATTCTGCCTCAGCCTCCCACAGGTGCACACCACCATGCCCAGTTATTTTGTATTTTTAGTACAGACAGGGT

TTCACCGCGTTGGCCAGGATCTCTAGATGT|TG|ACTCAGCCGGGCATGGTGGCTCACACCTGTAATCCCAACACTTCTGAGAGGTCGA
                               >chr1:29065847-29066118 - E=2e-151
GGAGGGCGGACCTCTTGAGCTCAGGAATTCAAGACCAGATTGGGCAACATAGTGAAACCCCCATCTCTACAAAAAAATTAAAAAAATTA

GCCGGGCATGGTGGTCCCAGCTACTTGGGAGGCTGAGGCGGGCAAAATCACCTGGTAGGCAGAGGTTGCAGTGAGCCGAGATTGCACCA

CTGCACTCCAGCCTGGGTGACAAGAGTTGACACTAC
```
